# Reduced striatal volumes in Parkinson’s disease: a magnetic resonance imaging study

**DOI:** 10.1186/2047-9158-1-17

**Published:** 2012-08-21

**Authors:** Toni L Pitcher, Tracy R Melzer, Michael R MacAskill, Charlotte F Graham, Leslie Livingston, Ross J Keenan, Richard Watts, John C Dalrymple-Alford, Tim J Anderson

**Affiliations:** 1 Department of Medicine, University of Otago, Christchurch, New Zealand; 2 New Zealand Brain Research Institute, 66 Stewart St, Christchurch 8011, New Zealand; 3 Department of Psychology, University of Canterbury, Christchurch, New Zealand; 4 College of Medicine, University of Vermont, Burlington, VT, USA; 5 Christchurch Radiology Group, Christchurch, New Zealand; 6 Department of Neurology, Canterbury District Health Board, Christchurch, New Zealand

**Keywords:** Magnetic resonance imaging, Volumetry, Caudate, Putamen, Parkinson’s disease

## Abstract

**Background:**

The presence and extent of structural changes in the brain as a consequence of Parkinson’s disease (PD) is still poorly understood.

**Methods:**

High-resolution 3-tesla T1-weighted structural magnetic resonance images in sixty-five PD and 27 age-matched healthy control participants were examined. Putamen, caudate, and intracranial volumes were manually traced in the axial plane of 3D reconstructed images. Striatal nuclei volumes were normalized to intracranial volume for statistical comparison. Disease status was assessed using the Unified Parkinson’s Disease Rating Scale and Hoehn and Yahr scale. Cognitive status was assessed using global status tests and detailed neuropsychological testing.

**Results:**

Both caudate and putamen volumes were smaller in PD brains compared to controls after adjusting for age and gender. Caudate volumes were reduced by 11% (p = 0.001) and putamen volumes by 8.1% (p = 0.025). PD striatal volumes were not found to be significantly correlated with cognitive or motor decline.

**Conclusion:**

Small, but significant reductions in the volume of both the caudate and putamen occur in PD brains. These reductions are independent of the effects of age and gender, however the relation of these reductions to the functional loss of dopamine, which is characteristic of PD, remains unclear.

## Background

The striatum (caudate and putamen) is the major input structure of the basal ganglia complex and is an essential part of neural networks involved in motor and non-motor function [1]. Striatal function is severely impaired in Parkinson's disease (PD), which depletes the neuromodulatory influence of ventral midbrain dopamine-producing neurons on these circuits and disrupts the balance of multiple corticostriatal circuits.

It is, however, unclear whether PD also produces gross morphological (volumetric) changes in the striatum. PD manifests progressively worsening motor and non-motor (cognitive and behavioral) dysfunction, which may in part reflect anatomical changes at the level of the putamen (motor) and caudate (oculomotor, cognitive and behavioral). Previous magnetic resonance imaging (MRI) studies have variously reported decreased or non-significant volume differences for these striatal structures using manual or semi-automated tracing methods [2-8] (Table 1).

**Table 1:** Summary of studies investigating striatal and total brain volumes in Parkinson’s disease using manual tracing and semi-automated methods

**Study**	**Magnet strength**	**Slice thickness**	**N PD/Control**	**TBV/ICV (effect size)**	**CN (effect size)**	**PUT (effect size)**	**Other**
Lisanby. *et al.*, 1993. [6]	1.5 T	5.0 mm	21/21	-	↓ (−1.4)	↓ (−1.8)	↓ Thal
Schulz. *et al.*, 1999. [8]	1.5 T	0.9 mm	11/46	-	↔	↔	-
Ghaemi. *et al.*, 2002. [4]	1.0 T	2.0 mm	24/17	-	↔ (0.77)	↔ (0.34)	-
O’Neill. *et al.*, 2002. [7]	1.5 T	1.4 mm	10/13	↔ (0.41)	↔	↓ (−0.89)	↓ GP
Almeida. *et al.*, 2003. [2]	1.5 T	1.6 mm	28/35	↔ (0.003)	↔ (−0.02)	-	-
Krabbe. *et al.*, 2005. [5]	1.5 T	1.7 mm	21/19	↑ (0.47/0.75)	↔ (−0.08)	↓ (−0.99)	↓ SN
Geng. *et al.*, 2006. [3]	3.0 T	2.0 mm	16/8	↔ (−0.38)	↔ (−0.57)	↓ (−1.5)	↔ SN ↓ GP
Current study	3.0 T	1.0 mm	65/27	↔ (−0.13)	↓ (−0.80)	↓ (−0.52)	

Direction of difference: ↑ significant increase in volume, ↔ no significant change in volume, ↓ significant decrease in volume in PD compared to healthy controls. - Structure not investigated. Standardized effect sizes (in parentheses) were calculated where sufficient information was available. Negative values indicate PD volumes smaller than controls.

*TBV* total brain volume, *ICV* intracranial volume, *CN* caudate nucleus, *PUT* putamen, *Thal* thalamus, *GP* globus pallidus, *SN* substantia nigra pars compacta.

Geng *et al.*[3] decrease in GP between late PD (n = 8) and controls only. Krabbe *et al.*[5] ICV effect sizes represent the supratentorial and infratentorial (supra/infra in table) volumes which were measured separately.

We employed 3-tesla MRI to estimate *in vivo* volumes of the striatum, using a large sample that covered a wide range of the motor and cognitive changes evident in PD.

## Methods

### Participants

A total of 116 participants, 82 with a diagnosis of probable PD according to the UK Brain Bank criteria [9] and 34 healthy age-related controls were recruited as part of a continuing research programme. PD participants were recruited through a movement disorder clinic (TJA) and healthy controls were volunteers from the general population. Exclusion criteria were a history of another central nervous system disorder, such as stroke or head injury or a major depressive episode within the last six months. All participants underwent structural magnetic resonance scanning, clinical and cognitive assessment. Six (4 PD and 2 controls) did not meet the requirements of a standard magnetic resonance safety screen and were excluded. A further eighteen were subsequently excluded, five (3 PD and 2 controls) following clinical review of scans, three controls who met the criteria for mild cognitive impairment (MCI) and ten (all PD participants) because of the presence of lacunar infarcts or large perivascular spaces in the striatal or internal capsule regions (n = 6) or movement artefacts (n = 4). Demographic and clinical characteristics of the final cohort (PD n = 65 and control n = 27) included in this study are provided in Table 2. The Upper South Regional Ethics Committees, New Zealand, approved all procedures and all participants gave informed written consent.

**Table 2:** Demographic and clinical characteristics of study participants

	**PD (n = 65)**	**Control (n = 27)**
Male/female (n)	48/17	19/8
Age (years)	66.2 ± 9.4	68.7 ± 9.6
Education (years)	12.8 ± 2.9	13.4 ± 2.9
MMSE	28.0 ± 2.7	29.0 ± 1.1
MoCA	23.9 ± 5.1	27.0 ± 1.9
Symptom duration (years)	6.3 ± 5.7	
range	0.5-30	
UPDRS part III	33.7 ± 16.3	-
Modified H & Y stage (median)	2.5	
range	1–4	
Cognitive status (n)		
Unimpaired	41	27
MCI	14	
Dementia	10	
PD Medication Status (n)		
No medication	24	
On Medication	41	
LED (mg/day)	572.4 ± 339.5	

Values are expressed as mean ± standard deviation, unless otherwise stated. *MMSE* Mini Mental State Examination, *MoCA* Montreal Cognitive Assessment, *UPDRS* Unified Parkinson’s Disease Rating Scale, *H & Y* Hoehn and Yahr scale, *MCI* mild cognitive impairment, *LED* levodopa equivalent dose.

PD participants underwent assessment of PD symptom severity and disease stage using the Unified Parkinson’s Disease Rating Scale (UPDRS) [10] and the modified Hoehn and Yahr scale (H & Y) [11]. Participants were classified as having dementia, mild cognitive impairment (MCI) or being of normal cognition using the following battery of tests. Attention and working memory: digits forward and backward, digit ordering, map search, Trails A, Stroop colour and word naming; Executive function: letter, action and category fluency, and category switching, Trails B and Stroop interference; Visuospatial and perception: judgement of line orientation, fragmented letters and Rey-Osterrieth Complex Figure copy; Learning and memory: California Verbal Learning Test–Short Form and the Rey-Osterrieth Complex Figure Test. A dementia diagnosis followed the Movement Disorders Task Force criteria [12], requiring significant impairment in activities of daily living [13] and severe impairment (two or more standard deviations below normative data) in two or more cognitive domains. Mild cognitive impairment was defined as a score of 1.5 or more standard deviations below the normative score in at least two tests in one neuropsychological domain [14] and meets the Movement Disorders Task Force guidelines for MCI [15]. Global cognitive status was assessed with the Mini Mental State Examination (MMSE) [16] and Montreal Cognitive Assessment (MoCA) [17] (not available in 3 PD participants). The levodopa-equivalent dose (LED) for PD participants was calculated using previously published conversion factors [18-20].

### Image acquisition

Structural magnetic resonance images were acquired with a 3.0-tesla General Electric HDx scanner (GE Healthcare, Milwaukee, WI, USA) using a high-resolution 3D T1-weighted spoiled gradient recalled acquisition sequence: echo time = 2.8 ms; repetition time = 6.6 ms; inversion time = 400 ms; flip angle = 15°, slice thickness 1 mm, field of view = 250 x 250 mm^2^, acquisition matrix 256 x 256 and voxel size of 0.98 x 0.98 x 1 mm^3^. Reconstructed images with a matrix of 512 x 512 and voxel size 0.5 x 0.5 x 1 mm^3^ were used for the manual segmentation of regions of interest (ROI).

### Region of interest (ROI) manual tracing

**Figure 1:**
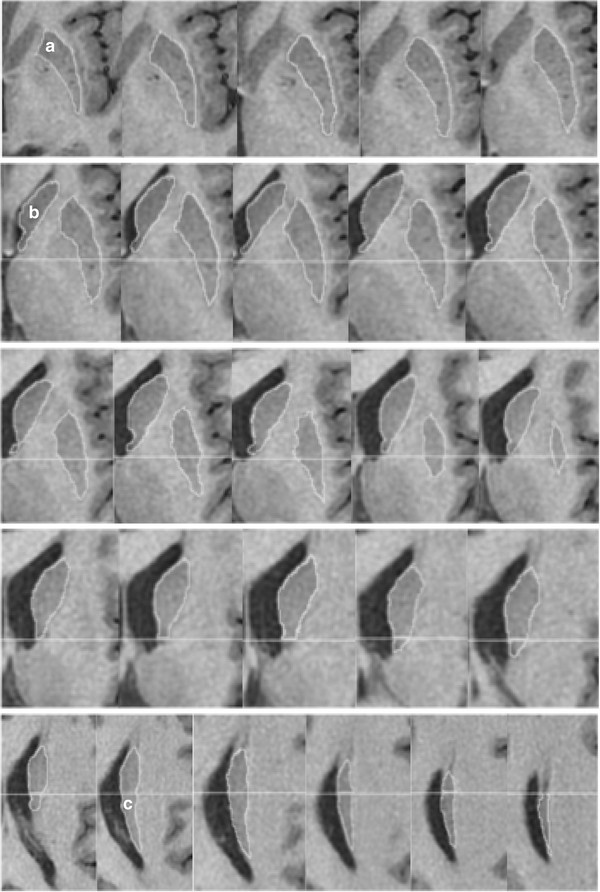
**Putamen and caudate volumes.** Continuous slices showing the full volumes of the left putamen and caudate in a brain of a Parkinson’s disease participant. a; putamen, b; caudate head, c; caudate body. Horizontal line; division between the caudate head and body. Line was defined in the coronal view at the level of the interventricular foramen.

Manual tracing of ROIs was performed by a single rater (TLP) using reconstructed images in MRIcroN [21] (http://www.mricro.com) implemented on a Windows PC. The rater was blinded to group membership. ROIs were traced in the axial plane with sagittal and coronal planes used for reference using the guidelines below. Each ROI was saved as a single volume, example tracings are shown in Figure 1.

#### Putamen ROI

Tracing of the putamen began in the first slice in which the putamen was clearly separated from the head of the caudate by the anterior limb of the internal capsule. Tracing continued on sequential slices for as long as a clearly distinguishable patch of grey matter was visible below the corona radiata. The globus pallidus and anterior limb of the internal capsule served as the medial border and the external capsule as the lateral border. Care was taken to ensure the claustrum was excluded.

#### Caudate nucleus ROI

The caudate was divided into two parts, head and body, which were traced separately. Tracing of the head of the caudate began in the first slice in which the internal capsule clearly separated the caudate from the putamen and in which the cavity of the lateral ventricle was visible. All gray matter below this slice was considered part of the nucleus accumbens. Small protuberances were included in the measurements of the head of the caudate as described by Looi *et al.*[22]. The boundary between the caudate head and body was defined, in the coronal view, as the first slice in which the interventricular foramen was present [23], i.e. when the cavities of the lateral and third ventricle were seen to be continuous. An ROI, covering the entire coronal slice at this level, was created and overlaid on the image, giving a horizontal line across the axial slice. Regions of caudate above to the line were designated the head, and regions below, designated the body. The medial border of the caudate was the cavity of the lateral ventricle. The lateral and superior borders of the caudate were the white matter of the internal capsule and corona radiata, respectively. The gray matter projections between the head of the caudate and the putamen, seen crossing the anterior limb of the internal capsule, were not included in the volume estimates of either nuclei. The caudate nucleus head and body were unable to be accurately delineated in six participants (PD n = 4, controls n = 2) due to the participants head position during scanning. Whole caudate nucleus volumes from these participants were included in the final analysis.

#### Intracranial volume

The intracranial volume (ICV) was manually traced in the axial view on every tenth section with intervening slices interpolated [24]. The ICV was traced between the foramen magnum and brain vertex. A starting slice within these boundaries was randomly selected for each brain. The outline was traced following the inner layer of the dura where visible, and the contour of the brain where dura was not visible. The venous sinuses and pituitary fossa were excluded from the ICV calculation.

### ROI volume calculation

A custom Matlab (The MathWorks Inc, Natick, USA) script was used to count the number of voxels in each ROI. This value was then multiplied by the reconstructed voxel size to give the ROI volume in mm^3^. Striatal volumes were normalized to the intracranial volume (bilateral structure volume/ICV x 100%) for statistical comparison.

### Statistics

Statistical analyses were conducted using SPSS 17 (SPSS Inc, Chicago) using normalized volumes (significance was set at p < 0.05). Analysis of covariance was used to compare ICV normalized volumes with group and gender as fixed factors and age as a covariate. Effect sizes were calculated from the estimated marginal means produced by the analysis of covariance. Associations between volumes, demographic and clinical variables were assessed using Pearson’s correlation statistic in a single matrix.

## Results

### Participant demographics

There was no difference in the age (F(1,91) = 1.39; p = 0.241) or years of education (F(1,91) = 0.947; p = 0.333) between PD and control groups (Table 2). There was a significant difference in MoCA scores (F(1,91) = 9.22; p = 0.003), but not MMSE scores (F(1,91) = 3.41; p = 0.068).

### ROI measurement reliability

The reliability of volumetric measurements was assessed by randomly selecting 15 scans, duplicating them and including them within the larger sample. The rater was blinded to this process. The reliability of ROI tracing was calculated using raw voxel size measurements from the duplicate scans and the intraclass coefficient statistic. Reliability coefficients were: right putamen, 0.99; left putamen, 0.96; right caudate head, 0.97; right caudate body, 0.87; left caudate head, 0.99; left caudate body, 0.97; and ICV, 0.99.

### ROI volumes

Intracranial volume did not differ between PD and healthy controls. (Control; mean ± sd. 1524.5 ± 177 cm^3^, PD; 1546.7 ± 159 cm^3^; F(1,91) = 0.35, p = 0.56, effect size = −0.13).

The volumes (mm^3^) of each ROI and of the total (left + right) putamen and caudate are shown in Table 3. To control for global differences in head size, striatal volumes were normalized to the ICV (see Table 3).

**Table 3:** Raw and normalized ROI volumes

	**PD (n = 65)**		**Control (n = 27)**	
**Brain region**	**Volume (mm**^**3**^**)**	**Normalized volume (%ICV)**	**Volume (mm**^**3**^**)**	**Normalized volume (%ICV)**
R Putamen	3200.9 ± 600.3	0.208 ± 0.039	3301.3 ± 604.0	0.219 ± 0.045
L Putamen	3238.0 ± 560.0	0.210 ± 0.035	3409.1 ± 663.2	0.226 ± 0.050
R Caudate Head	2680.6 ± 436.5	0.173 ± 0.025	2739.8 ± 409.8	0.183 ± 0.032
R Caudate Body	577.4 ± 176.0	0.037 ± 0.011	616.6 ± 210.7	0.041 ± 0.013
L Caudate Head	2627.4 ± 427.2	0.170 ± 0.026	2697.6 ± 357.41	0.180 ± 0.030
L Caudate Body	530.5 ± 170.3	0.034 ± 0.010	577.2 ± 167.9	0.038 ± 0.009
Total R Caudate	3226.0 ± 534.7	0.210 ± 0.030	3490.3 ± 686.3	0.230 ± 0.041
Total L Caudate	3120.8 ± 541.8	0.202 ± 0.031	3392.6 ± 587.1	0.224 ± 0.036
Total Putamen (L + R)	6438.9 ± 1092.4	0.418 ± 0.066	6710.5 ± 1239.6	0.445 ± 0.093
Total Caudate (L + R)	6346.8 ± 1059.2	0.411 ± 0.067	6882.9 ± 1250.4	0.454 ± 0.076
ICV	1,546,729.1 ± 159,539.4		1,524,478.5 ± 177,354.4	

*ICV* intracranial volume, *L* left, *R* right, *PD* Parkinson’s disease.

Analysis of normalized striatal nuclei volumes indicated that both the caudate and putamen were smaller in PD compared to controls (Table 4), representing a 11% volume reduction in the caudate and an 8.1% volume reduction in the putamen. The model controlled for gender and age, with marginal means calculated assuming an age of 67 years, the average for the entire sample. Although both the caudate head and body showed reductions in volume only the head volume was significantly smaller in PD compared to controls (Table 4).

**Table 4:** Results of striatal volume comparisons

**Striatal Region**^**ψ**^	**PD (n = 65)**	**Healthy Controls (n = 27)**	**F(1,91)**	**p**	**Effect size**
Normalized Caudate	0.416% (0.400 – 0.433)	0.468% (0.443 – 0.493)	11.38	0.001	−0.80
Normalized Putamen	0.422% (0.405 – 0.440)	0.459% (0.432 – 0.442)	5.22	0.03	−0.52
**Gender effects**^**ψ**^	**Male (n = 67)**	**Female (n = 25)**	**F(1,91)**	**p**	**Effect size**
Normalized Caudate	0.423% (0.407 – 0.439)	0.461% (0.435 – 0.487)	6.2	0.02	−0.59
Normalized Putamen	0.425% (0.408 – 0.442)	0.456% (0.429 – 0.483)	3.8	0.05	−0.47
**Caudate sub-regions**^**ζ**^	**PD (n = 58)**	**Healthy Controls (n = 25)**	**F(1,82)**	**p**	**Effect size**
Normalized Head	0.351% (0.338 – 0.363)	0.381% (0.363 – 0.399)	7.4	0.008	−0.66
Normalized Body	0.071% (0.064 – 0.077)	0.078% (0.070 – 0.086)	2.0	0.16	−0.32
Cognitively normal comparison^*^	**PD normal cognition (n = 41)**	**Healthy Controls (n = 27)**	**F(1,67)**	**p**	**Effect size**
Normalized Caudate	0.424% (0.401 – 0.446)	0.470% (0.442 – 0.497)	6.0	0.02	−0.65
Normalized Putamen	0.429% (0.406 – 0.453)	0.465% (0.437 – 0.494)	3.9	0.05	−0.48

Values are estimated marginal means (95% confidence intervals) calculated from analysis controlling for gender and age.

^ψ^Assumed age of 67 years; ^ζ^Assumed age of 66.4 years; ^*^Assumed age of 65.7 years.

Both age and gender were found to have significant effects on striatal nuclei volume. Age effects were greatest: caudate; F(1,91) = 14.6, p < 0.001 and putamen; F(1,91) = 39.5, p < 0.001. Regression analysis estimated the volume losses to be 28 mm^3^/year in the caudate and 57 mm^3^/year in the putamen. The gender effect indicated that females had larger striatal volumes, with the caudate but not the putamen reaching significance (see Table 4). There were no group by gender interactions: caudate; F(1,91) = 0.325, p = 0.57, putamen; F(1,91) = 0.01, p = 0.92.

Antiparkinsonian medications were not found to have an effect on striatal size: caudate; F(1,64) = 0.30, p = 0.56, putamen; F(1,64) = 2.66, p = 0.11.

### Effect of clinical and cognitive measures

**Figure 2:**
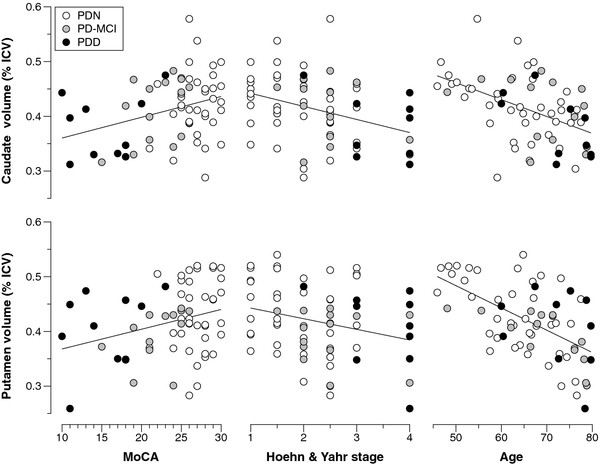
**Association between striatal volumes and clinical measures.** Each circle represents one Parkinson’s disease patient, either with normal cognition (PDN), mild cognitive impairment (PD-MCI), or dementia (PDD). Left column: The volume of each striatal structure was positively associated with overall cognitive status, as assessed by the Montreal Cognitive Assessment (MoCA, maximum score = 30). Middle column: Both structures decreased in volume with increasing motor disease severity, as measured by the Hoehn & Yahr scale. Right column: The volume of both structures decreased with age. The striatal associations with both MoCA and Hoehn & Yahr did not survive correction for age. The clear collinearity of the predictors, however, (i.e., older patients were more likely to be cognitively impaired and to have worse motor impairment) makes the attribution of causality difficult. Striatal volumes are expressed as percentage of intracranial volume (%ICV).

Effect sizes slightly smaller than those obtained in the full sample comparison were obtained when comparing cognitively normal PD participants (n = 41) to healthy controls, although the putamen no longer reached significance (see Table 4). There was no significant difference in striatal volumes between the PD cognitive groups (Caudate: PD-normal, 0.419 ± 0.060; PD-MCI, 0.411 ± 0.058; PDD, 0.380 ± 0.057. F(1,64) = 0.64, p = 0.53. Putamen: PD-normal, 0.429 ± 0.068; PD-MCI, 0.393 ± 0.048; PDD, 0.407 ± 0.071. F(1,64) = 0.99, p = 0.38). There were no significant differences in striatal volumes between patients with the initial onset of motor symptoms on the left versus the right (analysis restricted to early stage PD; H & Y stage 1 to 2.5 and cognitively intact). There was no association between striatal volumes and total UPDRS part III motor score. There was also no association when the UPDRS scores were separated into left and right-sided items and correlated with the contralateral striatal volumes. There was an overall correlation between striatal volumes and H & Y stage (Figure 2: caudate: r = −0.36, p = 0.004 and putamen: r = −0.27, p = 0.03). The variability within each H & Y stage meant that there were no significant differences between individual stages. Striatal volumes were also correlated with MoCA scores (Figure 2: caudate: r = 0.32, p = 0.010 and putamen: r = 0.289, p = 0.02) but not MMSE or disease duration.

Given the significant influence of age and gender on striatal volumes the significant correlations were retested using partial correlational analyses controlling for these factors, following which, no significant associations were observed (caudate: H & Y stage; r = −0.20, p = 0.13. MoCA; r = 0.11, p = 039, and putamen: H & Y; r = 0.00, p = 1.0, MoCA; r = 0.03, p = 0.80). The association between striatal volumes and age is shown in Figure 2. Because age, cognitive status and disease severity are highly correlated with each other, it is difficult to determine which factors are primarily driving the association with striatal volume.

## Discussion

Decreased volumes of both the caudate and putamen were found in PD brains compared to healthy controls. This study provides the first 3-tesla evidence of caudate atrophy in PD brains, using manual tracing methods. The present findings contrast with prior studies utilising fewer patients and in which, reduced putamen volumes but not caudate volumes were reported [2-5,7,8]. A possible source of variation in the findings across previous studies is variation in imaging parameters, including magnet strength, scanning sequence, slice thickness and inter-slice gap. The present study has the advantage of enhanced image resolution offered by acquisition at 3-tesla compared to 1.5-tesla, and 1 mm-thick slices with no inter slice gap, enabling the full extent of the regions of interest to be viewed. Another potential source of variation may be the boundaries used to delineate each structure. Although the putamen and caudate have well-defined boundaries due to the surrounding white matter and lateral ventricles, these structures fuse anteriorly with the ventral striatum. Unfortunately, most previous studies do not give detailed descriptions of the boundaries used.

A volume reduction in the putamen has previously been accepted due to the reduced dopaminergic activity in the nucleus in early disease stages [3,4,7], but little attention has been given to potential mechanisms of such a reduction. Although MRI is not able to directly inform us on such mechanisms, post-mortem studies provide some insight. A reduction in the dendritic spine density and a shortening of dendritic length in the medium spiny neurons of the striatum [25,26], and frank loss of striatal cells [27] have been described in post-mortem tissue. Such changes may contribute to a volume reduction and could significantly influence the functionality of the vast cortical projections to the striatum, which synapse on the medium spiny neuron dendritic arbour. Whether these events occur as a direct result of dopamine loss is unclear.

The volume reduction in the caudate was greatest in the head of the nucleus with non-significant volume changes in the body. The head of the caudate is more involved in cognitive function than the body, which is primarily involved in oculomotor function. However, our results do not suggest that the volume loss in the caudate is associated with cognitive status, as we also observed reduced caudate volume when comparing the cognitively unimpaired PD participants to healthy controls and no significant changes in volume between the PD cognitive groups. Whether caudate volume loss contributes to an increased susceptibility to later cognitive decline remains unknown.

Aside from Ghaemi *et al.*[4] all earlier studies from which we were able to calculate effect sizes indicated the putamen and caudate volumes to be smaller in PD brains compared to control brains (Table 1). The effect size for the caudate volume decrease in the study by Geng *et al.*[3] was of a reasonable size, but no significant reduction in PD volume was reported, suggesting that the study may have been underpowered to detect the difference in this measurement. Given the consistency in direction of effect sizes across previous studies, and the large sample used in our study, we believe our results reflect true changes in striatal volume in PD.

The absence of an effect of laterality of symptoms (whether side of initial onset or currently most affected side) upon striatal volume is consistent with previous studies [3]. The lack of association between striatal volumes and disease stage indicates that continued loss of volume over time due to disease processes may be of a relatively small magnitude and unable to be teased apart from the losses associated with increasing age. This is in contrast to the previously reported negative association between putamen volume and H & Y stage [3]. Since H & Y stage was significantly correlated with age in this current study, and the correlation between H & Y stage and volumes was not present after correcting for age, the association reported previously may have been, at least in part, due to age rather than more advanced stages of PD. Further investigation into the relationship between disease stage and striatal volume would require greater numbers of younger people with more advanced symptoms. Nigro-striatal neuronal loss is approximately 60% by the time of symptom onset [28] with rates of loss greater in earlier rather than late stages of the disease. Thus, it should not be surprising that we observed a significant reduction in striatal volumes in patients with normal cognition and no significant change with advancing disease (as measured by H & Y stage).

A limitation to the study is the unbalanced sample sizes in the cognitive sub-groups. Increased numbers of MCI and dementia PD participants would strengthen the analysis based on cognitive status. Also, improved delineation of the sub-regions of the caudate may be achieved in the future with the utilisation of diffusion tensor imaging tractography.

In summary, we have provided evidence at 3-tesla, that the caudate and putamen undergo volume loss in Parkinson’s disease, even early in the disease course. These new data, in conjunction with the existing literature, confirm the presence of atrophy in the striatum in PD brains over and above the influence of natural aging, although not with declining cognitive function and increasing motor impairment. Given the overlap in volumes across groups, such measures would not be useful as a diagnostic tool, however, knowledge of such volume reductions is important for understanding disease effects on the brain.

## Abbreviations

PD: Parkinson’s disease; MCI: Mild cognitive impairment; PDD: Parkinson’s disease with dementia; UPDRS: Unified parkinson’s disease rating scale; H & Y: Hoehn and yahr scale; LED: Levodopa-equivalent dose; ROI: Region of interest; ICV: Intracranial volume; MMSE: Mini mental state exam; MoCA: Montreal cognitive assessment.

## Competing interests

The authors declare no competing interests.

## Authors’ contributions

TLP - Project: conception and execution; Statistical analysis: design and execution; Manuscript: writing of first draft; TRM – Project: conception and execution; Manuscript: review and critique; MRM – Project: conception; Statistical analysis: review and critique; Manuscript: review and critique; CFG – Project: execution; Manuscript: review and critique; LL - Project: organization and execution; Manuscript: review and critique; RK - Project: execution; Manuscript: review and critique; RW- Project: organization and execution; Manuscript: review and critique; JCD-A - Project: conception, execution; Statistical analysis: review and critique; Manuscript: review and critique; TJA - Project: conception, execution; Manuscript: review and critique. All authors read and approved the final manuscript.
